# Fluorescence lifetime imaging through scattering media

**DOI:** 10.1038/s41598-023-30055-7

**Published:** 2023-02-21

**Authors:** Sebastian Nilsson, Elias Kristensson, Marcus Aldén, Joakim Bood, Andreas Ehn

**Affiliations:** Combustion Physics, Department of Physics, Faculty of Engineering, Lund University, Professorsgatan 1, 223 63 Lund, Sweden

**Keywords:** Imaging and sensing, Atomic and molecular interactions with photons

## Abstract

Fluorescence lifetime determination has proven to be useful, e.g. identification of molecules, quantitative estimation of species concentration and determination of temperatures. Lifetime determination of exponentially decaying signals is challenging if signals of different decay rates are being mixed, resulting in erroneous results. Such issues occur when the contrast of the measurement object is low, which can be limiting in applied measurements due to spurious light scattering. A solution is presented here where structured illumination is used to enhance image contrast in fluorescence lifetime wide-field imaging. Lifetime imaging determination was carried out using Dual Imaging Modeling Evaluation (DIME), and spatial lock-in analysis was used for removing spurious scattered signal to enable fluorescence lifetime imaging through scattering media.

## Introduction

Fluorescence lifetime imaging (FLI) is an optical measurement technique that determines spatially resolved lifetime distributions of fluorescing species. The fluorescence decay rate of an ensemble of excited molecules is dependent on the different loss mechanisms that act on the excited molecules. These loss mechanisms’ rates depend on parameters such as temperature, pressure, and number density of colliding partners. Hence it is possible to obtain information about temperature and species concentration by determining a fluorescence lifetime distribution^1^. Even though it is a general approach, FLI is most commonly applied in life-science microscopy^2–4^. However, the experimental schemes and ideas have also been used in numerous scientific fields, such as art conservation^5,6^, combustion^7,8^ and remote sensing^9,10^.

A mode-locked laser with pulse duration in the picosecond regime is sufficiently short to resolve typical fluorescence lifetimes in the time domain^1^. The bandwidth of such laser pulses is also narrow enough to provide selective species excitation. Temporally averaged wide-field FLI of stationary targets can be achieved by combining such an excitation approach with time-gated detection. In contrast, dynamic events require instantaneous imaging and are effectively captured with a dual time-gated detector setup^11^. The time gates of these two cameras have different temporal characteristics yielding two views of the same target but in different time windows (integration times). A group of lifetime evaluation methods, based on the Rapid Lifetime Determination (RLD) algorithm^12,13^, account for these acquisition characteristics to utilise differences in the two images that can be used to form an instantaneous two-dimensional lifetime image. However, obtaining accurate lifetime images based on pixel intensity ratios requires high-fidelity images with high contrast free from interfering signal contributions. Such optimal conditions are rarely achievable in practical applications due to spurious scattering.

**Figure 1:**
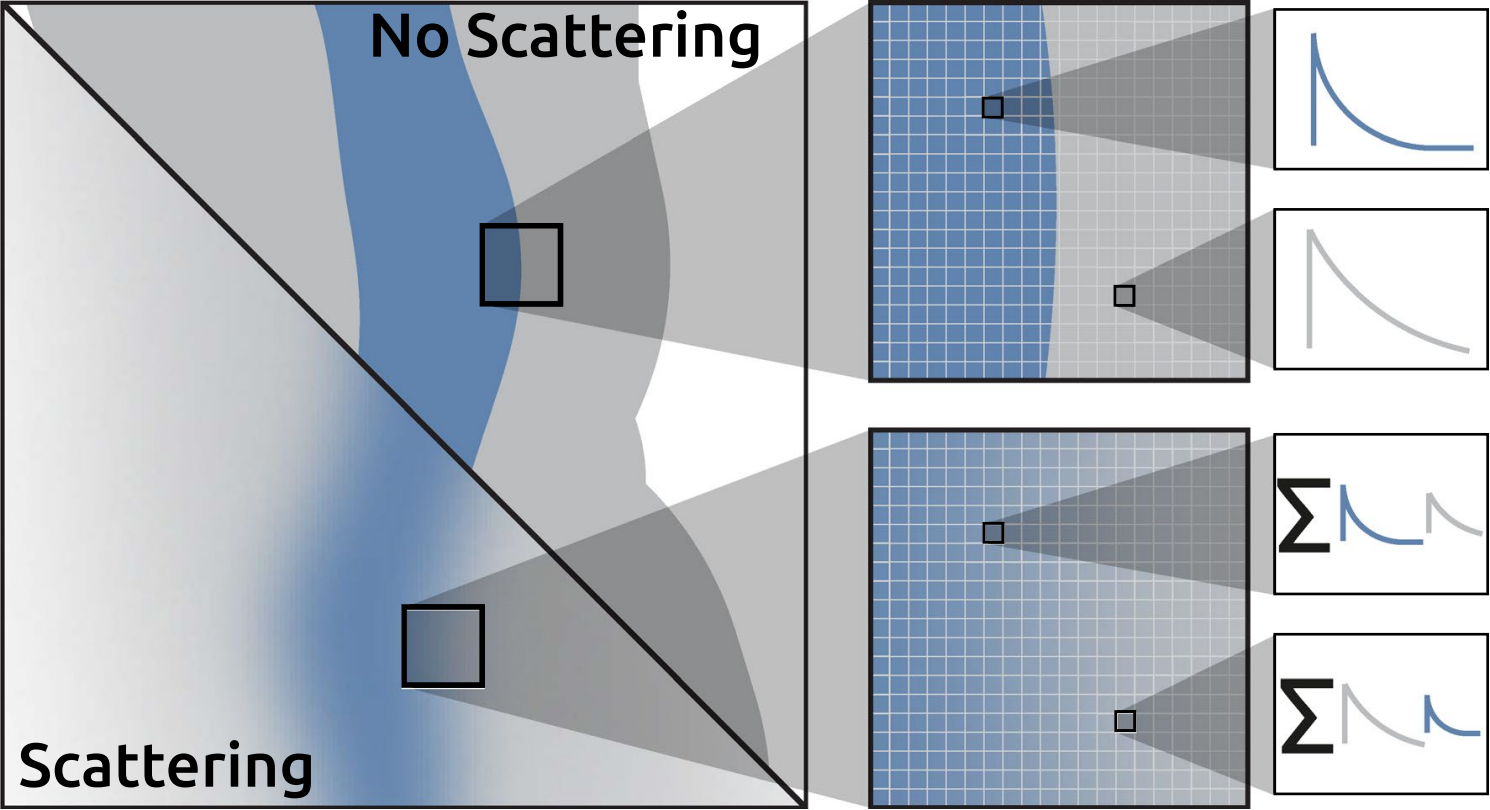
The upper part shows a case where there is no scattering, the short (blue) and long (gray) fluorescence lifetimes in the detector plane can be correlated to a specific location in the image plane. With the effects of scattering shown in the lower part of the figure, the contribution from nearby areas in the image plane will contribute to the detected luminescence lifetime in the detector plane such that the spatial resolution is decreased. The signal in these pixels will be a sum of signals from nearby locations.

While non-laser-induced background signals often can be estimated and subtracted, signal contributions from spurious scattered light cannot and need to be approached differently. Spurious scattered light is a significant issue for studies of sprays, both when (i) the laser interacts with the spray and (ii) the laser-induced signal propagates towards the detector. To suppress this multiply scattered light, methods based on structured illumination have been developed^14–16^. The idea with such approaches is that photons that have experienced multiple scattering events will lose the superimposed structured information, in contrast to the singly scattered light. The contribution from singly scattered light can be extracted using a spatial frequency lock-in filtering algorithm^17–19^. For spray diagnostics, structured illumination is used to suppress multiply scattered light that blurs the image of liquid structures. In contrast, signals stemming from different areas in the image may have other lifetimes in FLI; see Fig. 1. Thus, multiple light scattering causes image blur and the mixing of signals with different lifetimes. The evaluated lifetime in a pixel with such mixed signals would not represent the lifetime in the corresponding image plane location.

In the current work, we investigate whether the structured illumination methodology in combination with spatial lock-in analysis can suppress the multiply scattered light to enable lifetime imaging in harsh environments. The fluorescence-lifetime images are obtained by Dual Imaging Modelling Evaluation (DIME), proposed by Ehn et al.^7,20,21^. The results show that lock-in can effectively suppress the scattering such that accurate lifetime images can be obtained with the ability to resolve sub-nanosecond fluorescent lifetimes. The results show great potential for FLI investigations of gas- as well as liquid- and solid phases in a scattering environment.

## Methods

Laser-induced fluorescence (LIF) images captured in harsh environments must first be corrected for scattering and interfering signals using structured illumination with lock-in analysis before accurate FLI images can be obtained. Structured illumination can be achieved by periodically modulating the laser sheet in the probe volume with a cosine wave^14,22^. Illuminating a fluorescing sample with such a modulated laser sheet will yield a LIF image, *I*, that is the product of the modulated laser intensity distribution and the spatial distribution of the molecule of interest, according to:

1 $$\begin{aligned} I = I_C + I_S \cdot \cos (2\pi \nu _{y} \cdot y + \phi ) \end{aligned}$$

where $\nu _y$ is the spatial modulation frequency in the *y*-direction, $\phi$ is an arbitrary spatial phase. This description involves two images, $I_C$ and $I_S$; $I_C$ is the conventional image representing the image one would obtain if the laser sheet is not modulated, whereas $I_S$ contains the amplitude of the modulation, which correlates to the intensity from the laser-induced fluorescence signal. The information in $I_S$ can be obtained by a frequency-sensitive lock-in analysis that separates the modulated signal from the non-modulated background ($I_C - I_S$)^17,19^. This analysis utilises that the modulated information is frequency-shifted to higher spatial frequencies in the Fourier domain. The information contained in the high-frequency components can be extracted by (i) filtering out all other information in the Fourier domain with a frequency filter, (ii) translating the filtered data to the origin of the Fourier domain, and (iii) inverse Fourier transforming the signal back to the spatial domain which results in a non-modulated image. These images that are now corrected for background- and scattering signals can now be analysed using DIME.

**Figure 2:**
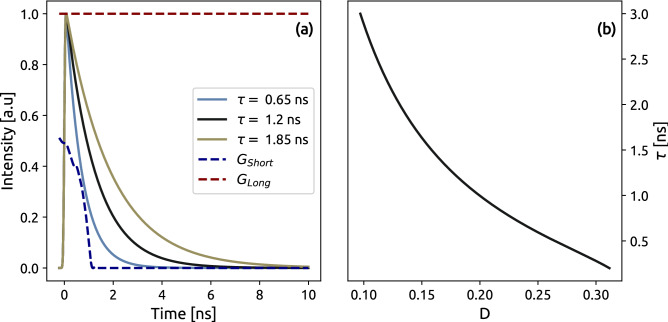
Simulated data, which forms the model for the DIME method. (**a**) Shows mono-exponential decay curves together with the gate functions for the long and short gates. Note that only the closing part of the short gate function is displayed, when the camera captures signal. (**b**) is the model of the simulated data using Eqs. (2) and (3), which is used to map an intensity ratio to a lifetime.

Rapid lifetime determination algorithms typically utilise two LIF images where each pixel in the images corresponds to the same point in the image plane. Each image is acquired with different gate characteristics that capture different parts of the fluorescence lifetime decay curve. The two gate functions that are used in this study are displayed in Fig. 2a, where the $G_{Long}$ captures the entire signal, and $G_{Short}$ captures the early part of the signal which yields the images $I_{Long}(x,y)$ and $I_{Short}(x,y)$, respectively. A ratio image can be formed between these images where the ratio value depends on the fluorescence decay time. When performing FLI with DIME this ratio image, *D*(*x*, *y*), is formed between $I_{Short}(x,y)$ and the sum of $I_{Short}(x,y)$ and $I_{Long}(x,y)$:

2 $$\begin{aligned} D = \frac{I_{Short}}{I_{Short}+I_{Long}}. \end{aligned}$$

To couple an experimental ratio image, *D*, to fluorescence lifetimes, a detection system model is created using the known time gate functions $G_j(t)$ and the temporally decaying signal *S*(*t*). Therefore, the detected intensity in each camera can be simulated using Eq. (3).

3 $$\begin{aligned} I_j = \int G_j(t)S(t)dt. \end{aligned}$$

**Figure 3:**
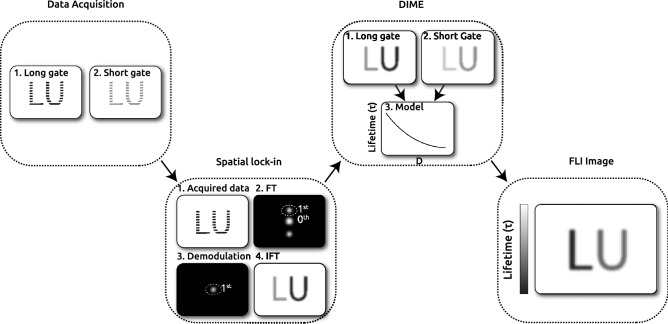
Flowchart of the methods applied in this study with graphical visualization of each method. (i) Data for long and short images were acquired during the experiment. (ii) The long and short images were analyzed using the spatial lock-in method to suppress scattering and remove the background. (iii) The DIME model is applied to the filtered images to extract (iv), the scattering and background-free FLI image.

In this investigation, *S*(*t*) is a mono-exponential function and gate functions, with the index *j*, are either the gate with the long or the short gate. A constant represents the long gate dashed red line in Fig 2a, because the rising and falling parts are well outside the time window of the LIF signal. Simulating the relative detected intensities for a range of fluorescence lifetimes using Eqs. (2) and (3) generate a function that can correlate a ratio to a unique lifetime. This function, $\tau (D)$, displayed in Fig. 2b, can then be used to convert the image ratio *D*(*x*, *y*) to a lifetime image, $\tau (x,y)$. A more detailed description of the DIME evaluation algorithm and a review of the experimental considerations can be found in^20^. This procedure for extracting fluorescence lifetime images captured in a scattering environment is graphically displayed in Fig. 3.

## Experimental Setup

**Figure 4:**
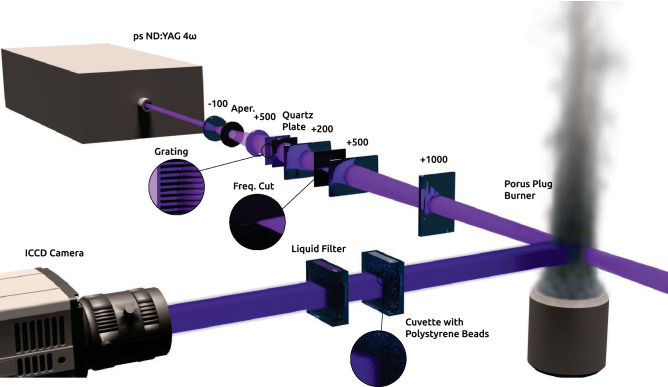
Schematic image of the experimental setup, all units are in mm. A water-filled quartz cuvette is located between the gaseous flow and the ICCD camera. The cuvette was seeded with polystyrene spheres to introduce scattering in the imaging system.

To demonstrate the capabilities of the methods presented in this paper, a flow system consisting of a stable laminar jet and co-flow of toluene-seeded gas mixtures with different amounts of $O_2$ was studied, as shown in the right part of Fig. 4. The vertically directed jet flow of toluene-seeded air was positioned at the center of the porous plug. The co-flow of toluene-seeded nitrogen-enriched air was fed through a porous plug to stabilize the laminar jet flow which allowed for image accumulation to provide clear high-fidelity data with good signal-to-noise ratio. The rather large difference in signal intensity between the two measurements required three times as many accumulations (3000) when imaging was performed through the scattering medium. This difference was accounted for, in the data analysis, as signal relative signal intensities are compared. Both gas mixtures were seeded with toluene by passing them through bubblers filled with liquid toluene. This experimental arrangement yields an FLI image with three zones where different lifetimes exist. The zone where the central jet is located should display a lifetime of 0.65 ns, whereas the zone that corresponds to the co-flow region should have a lifetime of 1.8 ns, according to previous work^23^. The third zone is the transition between the jet and the co-flow and should display lifetimes from 0.65–1.8 ns due to diffusion mixing.

The excitation source, shown to the left in Fig. 4, was a mode-locked Nd:YAG laser (Ekspla, PL2143C), which generates frequency quadrupled (266 nm) laser pulses with a pulse duration of 30 ps at a repetition rate of 10 Hz.

The laser beam was first expanded, cropped and collimated to provide a top hat-like profile for even illumination of the probe volume. The collimated beam was tagged with spatial frequency by transmission through a Ronchi grating. The Fourier components of the horizontally modulated structure were imaged onto a mask, using two cylindrical lenses with the power direction vertically oriented, that transmitted the $\pm 1{\text {st}}$ order frequency components. All other orders were blocked. A vertically oriented laser sheet was formed using a cylindrical lens focused at the center of the toluene-seeded gas jet. The quartz plate was used to shift the phase of the spatial modulation such that the conventional image could be retrieved^22^.

An ICCD camera (PI-MAX 3, Princeton Instruments) with a short gate option was used to detect the PLIF signal. The camera was equipped with a gen-II chip to provide high sensitivity in the UV region where toluene emits around 300 nm. The ICCD camera was equipped with a UV lens (UV-Nikkor f = 105 mm, f/4.5). A 20-mm quartz cuvette filled with dimethylformamide was positioned in front of the UV lens to suppress elastically scattered light. The first of the two measurements were conducted with a cuvette filled with deionized water in front of the ICCD camera. The gate function of the camera intensifier was mapped with this configuration by sequentially stepping the delay time as Rayleigh scattering was detected (without the filter). The short gate was set to 2.74 ns and the long gate to 500 ns. Polystyrene spheres were added to the water in a second experiment, forming a dense scattering medium between the measurement object and the detector. Fluorescence images were acquired with the short and long gates creating a set of two images.

## Result and discussion

**Figure 5:**
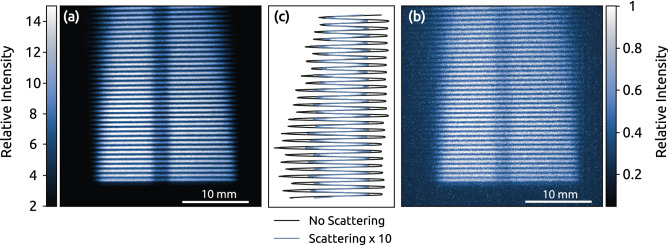
Raw data PLIF images (**a**) without additional scattering yields a contrast of M $\approx$ 0.44. (**b**) Imaging through scattering media (OD $\approx$ 1) reduces the contrast, M $\approx$ 0.37. (**c**) Cross sectional data, from the central radial position, display the difference in modulation for figure (**a**) and (**b**). Note that the signal intensity is about one order of magnitude lower when signal is collected though a scattering medium.

Experiments were performed with and without the scattering medium in the detection line, and typical results from these experiments display accumulated PLIF-images in Fig. 5a,b, respectively. The modulation pattern was visible in both images, and the gas jet, located at the center of the image, was slightly darker than the surrounding gas signal because the toluene-seeded air had a shorter lifetime, which reduced the fluorescence signal. The additional scattering introduced by the polystyrene spheres (i) deteriorates the image contrast and (ii) lowers the signal intensity because the light is scattered off.

Both effects are shown in Fig. 5c, where the black and blue curves display the data without and with scattering, respectively. The curves show the cross-sectional data at the zero-radial coordinates of the images in Fig. 5a,b, where the intensity difference is observed. The Michelson contrast is commonly used to define contrasts in images, where 1 is the maximum contrast and 0 is an image with equal intensity. The Michelson contrast of the measurement data captured without additional scattering is M $\approx$ 0.44, whereas additional scattering lowered the contrast to M $\approx$ 0.37. The modulation intensity was reduced by approximately one order of magnitude, indicating that the dense scattering medium had an optical density of approximately one.

**Figure 6:**
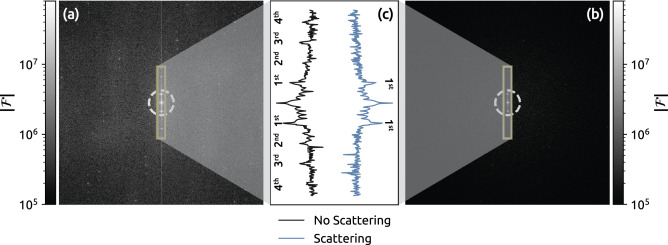
Fourier transform of the images shown in Fig. 5. (**a**) Shows data from the reference case (without scattering), whereas the data in (**b**) was acquired with additional scattering. (**c**) Display cross-sectional data from a rectangle in (**a**) and (**b**). The white dashed circles in both (**a**) and (**b**) represent the Fourier transformed PSF at three $\sigma$ (where the value reaches $e^{-4.5} \approx$ 1% of the maximum value).

The data that are spatially resolved in Fig. 5 were further investigated and compared in the Fourier domain, and the results are shown in Fig. 6. In Fig. 6a, the 1st order modulation shift can be seen along the center of the image in the vertical direction, and higher-order frequencies are shifted even further out in the reciprocal domain. However, these higher-order frequency components appear to disappear when the image is acquired through the scattering medium, as shown in Fig. 6b. This low-frequency filtering can be mathematically described by viewing the imaging transmission through the scattering medium as a convolution with a point spread function (PSF). For simplicity, it can be assumed that the PSF is a Gaussian kernel, because the Fourier transform of a Gaussian is Gaussian. The main difference between these two Gaussian’s is that the standard deviation ($\sigma$) in space is inversely proportional to the standard deviation in the frequency domain ($\sigma ^{-1}$), see Eq. (4).

4 $$\begin{aligned} \frac{1}{\sigma \sqrt{2\pi }}\text {exp}\left( -\frac{x^{2}}{2\sigma ^{2}} \right) \overset{{\mathscr {F}}\left( x\rightarrow \omega \right) }{\rightarrow } \textrm{exp}\left( -\frac{\omega ^{2}\sigma ^{2}}{2} \right) \end{aligned}$$

Furthermore, the convolution between a spatial image and PSF can be mathematically described as a multiplication of the two images in the Fourier domain. The PSF, which is a Gaussian located around the origin, acts as a low-pass filter in the Fourier domain because the high-frequency outer parts of the image are dampened when they are multiplied by the outer part of the Gaussian. A wide Gaussian PSF in the spatial domain corresponds to a situation in which imaging is performed through a very dense scattering medium, which then acts as a very narrow low-pass filter. Similarly, a very sharp imaging system has a very narrow PSF, which in turn will be a very wide frequency filter and will only slightly lower the high-frequency components in the Fourier domain. The Fourier transform of the PSF produced by the scattering medium in the current investigation was estimated from the relative intensity change that was seen without and with scattering, as shown in Fig. 6c. The frequency filter is illustrated in Fig. 6a,b using a white dashed ring with a radius of 3$\sigma$ from the origin.

Moreover, a juxtaposition frequency components is compared in a line graph in Fig. 6c. The 2nd up to the 4th order components are distinguishable without additional scattering while these higher-order components are in the noise floor when the image was acquired through the scattering medium. The 0th and 1st order components are clearly distinguishable in both cases while the 1st order components in the scattering case have reduced intensity.

**Figure 7:**
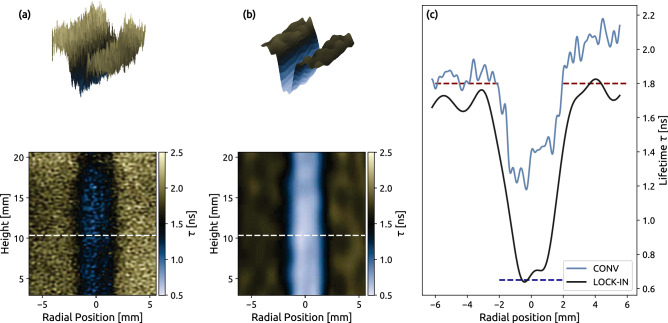
Fluorescence lifetime images for (**a**) conventional, (**b**) lock-in. The 3D plot shows the same data as its corresponding image. In (**c**), data were extracted along the dashed white line in each image and compared in a line plot. The red and blue dashed lines indicate expected values in the co-flow (red) and central jet (blue).

FLI images were produced using the conventional and lock-in methods using DIME, and their respective results are shown in Fig. 7. Both analysis methods display the desired characteristics, where the signal in the central jet has a shorter lifetime than in the co-flow. The lifetimes in the jet evaluated by the conventional analysis method, shown in Fig. 7a, are over-predicted by a factor of two compared to the expected lifetime. In the co-flow, where the lifetimes are expected to be symmetric around the jet, a slanted bias in the determined lifetime is observed in Fig. 7c. In contrast, applying structured illumination in conjunction with spatial lock-in analysis yields evaluated fluorescence lifetimes in good agreement with the expected values, as shown in Fig. 7b,c.

The over-prediction in lifetime determination for the conventional case is due to an inability to remove the background intensity offset central for all intensity-based methods which in a scattering environment yields a non-uniform background. Therefore, the background due to scattering can not be determined from any reference image or blank recording. Nevertheless, the average intensity offset in the conventional image was subtracted by the intensity value at the height of 3 mm, where no laser illumination occurred. However, this correction method is imperfect, and the residual background from scattering causes errors in the lifetime evaluation. Erroneous background subtraction also contributes to the over-prediction of the lifetime in the jet flow, but two additional mechanisms also contribute here. First, scattering causes the mixing of various signals in the probe volume, which means that the signal in each pixel will be a sum of exponential decays. This mixing of signals is illustrated in Fig. 1 and can be observed in Fig. 5 as a reduction in contrast between the two flows as scattering spheres are introduced. The effect of signal mixing due to scattering can be expressed as the sum of two exponential decays with different amplitudes, which is mathematically described as:

5 $$\begin{aligned} I_{pixel} = (1-\alpha )\cdot \exp {\bigg (-\frac{t}{\tau _1}\bigg )} + \alpha \cdot \exp {\bigg (-\frac{t}{\tau _2}\bigg )}. \end{aligned}$$

**Figure 8:**
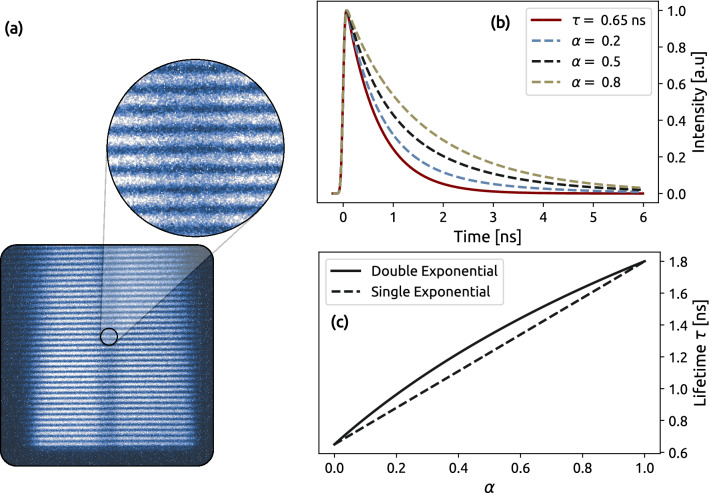
Determined lifetime by the DIME algorithm where (**a**) PLIF image of the toluene seeded flows display a deterioration of image contrast due to additional scattering, which causes signal mixing between jet- and co-flow. (**b**) Decay curves from mixed dual exponential decays show an increase in lifetime with higher mixing. (**c**) Dual exponential (dashed line) and a single exponential decay (solid line) with a mixing value of ($\alpha$) $\tau =(1-\alpha )\cdot \tau _1 + \alpha \cdot \tau _2$.

Parameter $\alpha$ ranges from 0 to 1 and describes how these signals are mixed. For example, the value $\alpha = 0.5$ corresponds to a situation in which the two lifetimes have equal contributions (initial peak intensity, corresponding to the number density of toluene). To investigate how signal mixing affects the DIME evaluation method, it was applied to Eq. (5) as the parameter $\alpha$ was scanned from 0 to 1, with $\tau _1 = 0.65$ ns and $\tau _2 = 1.8$ ns. The result from these simulations is displayed in Fig. 8 and shows that the lifetime determined by the DIME algorithm is always skewed to the longer lifetime when two signals are mixed. This overestimation is because the signal luminosity of an exponential decay curve is greater if the lifetime is longer; see Fig. 2a. Second, the over-prediction in the lifetime of the jet flow is the result of a much larger volume ($\sim$85%) being excited in the co-flow and, due to multiple light scattering, the fluorescence signal from this larger excited volume appears to originate from the jet, illustrated in Fig. 8a.

DIME based on structured illumination combined with lock-in analysis provides evaluated lifetimes, which agrees well with theoretical values for both flows. Also, the central jet becomes clearly distinguishable after the lock-in analysis, and the diffusion zone emerges where oxygen diffuses into the co-flow. This improvement is due to (i) the ability of structured illumination to remove the signal mixing caused by multiple light scattering (ii) and the automatic subtraction of the background.

It should be noted that the lock-in analysis utilises a frequency filter which will reduce the spatial resolution of the original image, see Fig. 7a,b. Consequently, the lock-in method should thus be cautiously applied to objects with delicate image structures. In harsh environments, such details and strong image gradients are generally difficult to capture due to non-ideal imaging conditions. In the current case, however, these details are lost due to signal scattering, and under such conditions, spatial lock-in analysis can be applied without further deterioration of the image resolution.

## Conclusion

In this paper, we demonstrate the first successful implementation of wide-field FLI in a harsh environment, where the strong signal scattering has been suppressed to retrieve accurate lifetimes successfully. Fluorescence lifetimes were determined by DIME, while scattering suppression was achieved by employing structured illumination in conjunction with spatial lock-in analysis. The presence of scattering (i) leads to a lower signal-to-noise ratio and image resolution, (ii) yields a non-uniform background and reduces image contrast and (iii) causes the mixing of signals from different locations in the image plane. Consequently, these issues reduce precision and accuracy in the determined lifetime, as well as the specificity of the signal origin. It was found that lifetime determination of signals containing different lifetimes is biased towards the longer lifetime, making it particularly problematic to accurately determine lifetimes in small regions with fluorescence lifetimes shorter than their surroundings. To conclude, the combination of the three concepts—DIME, structured illumination and lock-in analysis—shows excellent potential for wide-field FLI in harsh environments, opening up for quantitative FLI-based analysis of multi-phase reacting processes on micro- and macroscopic scales.

## Data Availability

The data that support the findings of this study are available from the corresponding author upon reasonable request.
